# Efficient Removal of Cu(II), Zn(II), and Cd(II) from Aqueous Solutions by a Mineral-Rich Biochar Derived from a Spent Mushroom (*Agaricus bisporus*) Substrate

**DOI:** 10.3390/ma14010035

**Published:** 2020-12-23

**Authors:** Guosheng Zhang, Na Liu, Yuan Luo, Haibo Zhang, Long Su, Kokyo Oh, Hongyan Cheng

**Affiliations:** 1 College of Resources and Environment, Shanxi Agricultural University, Jinzhong 030801, China; zhanggs@stu.sxau.edu.cn (G.Z.); liuna@sxau.edu.cn (N.L.); luoyuan@sxau.edu.cn (Y.L.); zhanghb@sxau.edu.cn (H.Z.); sulong@stu.sxau.edu.cn (L.S.); 2 Center for Environmental Science in Saitama, Kazo City, Saitama 347-0115, Japan; peace6728@yahoo.co.jp

**Keywords:** spent mushroom substrate, biochar, pyrolysis temperature, mineral, heavy metal, sorption characteristic, mechanisms

## Abstract

This study evaluated the novel application of a mineral-rich biochar derived from a spent *Agaricus bisporus* substrate (SAS). Biochars with various pyrolysis temperatures (350–750 °C) were used to remove Cu(II), Zn(II), and Cd(II) from aqueous solutions. The adsorption characteristics and removal mechanisms of the biochars were investigated. The adsorption kinetics and isotherm data were fitted well by pseudo-second-order and Freundlich models. The Langmuir maximum removal capacity (*Q*_max_) values of Cu(II), Zn(II), and Cd(II) were ordered as SAS750 > SAS350 > SAS550, and the *Q*_max_ values of SAS750 were 68.1, 55.2, and 64.8 mg·g^−1^, respectively. Overall, the removal mechanisms of biochar at a low production temperature (350 °C) to Cu(II), Zn(II), and Cd(II) were mainly via ion exchange (54.0, 56.0, and 43.0%), and at a moderate production temperature (550 °C), removal mechanisms were mainly via coordination with π electrons (38.3, 45.9, and 55.0%), while mineral precipitation (65.2, 44.4, and 76.3%, respectively) was the dominant mechanism at a high produced temperature (750 °C). The variation of the mutual effect of minerals and heavy metals was the predominant factor in the sorption mechanism of mineral precipitation and ion exchange. The results demonstrated that spent *Agaricus bisporus* substrate biochar is a potential candidate for the efficient removal of heavy metals, which provides a utilization route for spent mushroom substrates.

## 1. Introduction

Edible fungi are organic, green, and healthy foods. As one of the most promising food industries, the edible fungus industry has developed rapidly in recent decades. However, approximately 5 kg of spent mushroom substrate is generated during the production process of 1 kg of edible fungi [1,2]. In 2018, approximately 70 million tons of edible fungi were produced in China; hence, a large amount (approximately 350 million tons) of spent mushroom substrate had to be treated and utilized. Various methods have been proposed for the reuse of spent mushroom substrates, such as conventional treatment methods, e.g., as compost [3,4] or feed [5]; however, these methods are only suitable for treating small amounts of substrate. Most spent mushroom substrates are not effectively disposed of and are randomly stacked or burned in open air [6]. Therefore, the development of an economical and environmentally friendly treatment method for spent mushroom substrates is necessary.

Biochar is a carbon-rich solid material that is produced from biomass in an oxygen-limited atmosphere and can adsorb a variety of pollutants [7], especially in aqueous solutions that are polluted by heavy metals [8,9]. A variety of agricultural by-products have been converted into biochar and used as environmental adsorbents to remove heavy metals. Previous studies have reported that biochars derived from feedstock materials such as peanut shells [10,11], wood chips [12,13], corn straw [14,15], and rice straw [16,17] can remove Cu(II), Zn(II), and Cd(II) effectively from aqueous solutions. Recent reports have highlighted the indispensable role of mineral components in biochar sorption [18,19], and mineral-rich biochar has excellent removal ability for heavy metals. Biochar with a high mineral content can remove heavy metals through complex mechanisms, which may involve (1) mineral precipitation, (2) cation exchange, (3) complexation of surface functional groups, and (4) interaction of π electrons with heavy metals [20,21,22]. In summary, mineral-rich biochar may be an effective material for removing a variety of heavy metals from water and is more economical than other materials.

Mineral-rich biochar can be produced from spent *Agaricus bisporus* substrates. As a delicious mushroom, *Agaricus bisporus* is grown worldwide and loved by consumers. In the cultivation of *Agaricus bisporus*, in addition to composting and fermentation with chicken manure and rice straw, calcium-containing minerals are applied as nutrients and in surface-covering soil. These processes form a culture medium that is suitable for mycelial growth [5], which renders the spent *Agaricus bisporus* substrate rich in minerals and potentially suitable for the preparation of biochar. Few reports have been published on the production of biochar from spent *Agaricus bisporus* substrates. The properties of the prepared biochar, its performance, and its mechanism of adsorbing heavy metals have yet to be elucidated. Meanwhile, the contribution to the biochar removal mechanism of heavy metals is attributed to the constraints of the biochar source and the pyrolysis temperature [23]. The properties of biochar differ according to the pyrolysis temperature [24], thereby resulting in differences in importance among the mechanisms in the sorption process. Few current studies have clarified the relationship between the differences in physicochemical properties among biochars that were synthesized at different pyrolysis temperatures and the relative contributions of various mechanisms to the sorption of heavy metals. In addition, excessive heavy metals, namely, copper (Cu) and zinc (Zn), are present in livestock and poultry breeding wastewater due to feeding addition, and highly toxic cadmium (Cd) is also considered a potential environmental threat.

In this study, biochars were prepared from a spent *Agaricus bisporus* substrate at various pyrolysis temperatures (350–750 °C), and their physical and chemical properties were characterized via various techniques (1) to determine the ability of biochar to adsorb Cu(II), Zn(II), and Cd(II) in aqueous solutions; (2) to determine the effects of the pyrolysis temperature on the sorption capacity of the biochar; and (3) to qualitatively and quantitatively investigate the mechanism via which the biochar removes Cu(II), Zn(II) and Cd(II) from aqueous solutions.

## 2. Materials and Methods

### 2.1. Biochar Preparation

The feedstock of a spent *Agaricus bisporus* substrate (SAS) was provided by the Edible Fungus Center of Shanxi Agricultural University, Taigu County, China. The fresh feedstock was placed in a ventilated environment to air-dry for a week, and small stones were manually removed at this time. After air-drying, the SAS was crushed to a particle size of >0.5 cm using a crusher and dried at 60 °C until it reached a constant weight. Approximately 55–60 g of the dried SAS was placed in a porcelain crucible and compacted (with a volume of 100 mL), and the crucible was put in a muffle furnace with a limited oxygen environment. Then, the temperature was increased at a rate of 20 °C·min^−1^ to the specified temperature (350, 450, 550, 650, and 750 °C), and the sample was pyrolyzed at the peak temperature for 3 h. The spent *Agaricus bisporus* substrate biochars (SASCs) (the remaining solid substances after pyrolysis) were labeled as SAS350, SAS450, SAS550, SAS650, and SAS750 according to the pyrolysis temperature. All SASCs were ground to particle sizes of > 0.15 mm prior to use. Demineralized biochar samples were obtained by eluting the biochar with 1 mol·L^−1^ HCl and washed with distilled water until the pH of the solution no longer changed.

### 2.2. Characterization of SASCs That Were Obtained at Various Pyrolysis Temperatures

The pH values of all samples were determined using a pH meter (Lei-ci PHS-3Ct, Shanghai, China) with a water/sample ratio of 10:1 after shaking for 30 min. The concentrations of elements (C, N, H, and S) were determined via elemental analyses (Vario Macrocube Elementar, Langenselbold, Germany). The surface areas were determined from Brunauer-Emmett-Teller (BET) isotherms with N_2_ sorption at 77 K that were obtained using a surface area analyzer (ASAP2020, Micromeritics, Norcross, GA, USA). The ash contents of the SASCs were determined by heating the samples at 700 °C for 2 h, and the metal contents were determined via inductively coupled plasma optical emission spectrometer (ICP-OES, Optima 5300 DV, PerkinElmer, Waltham, MA, USA). The morphologies and elemental species of the biochars were analyzed using scanning electron microscopy and energy dispersive X-ray (SEM/EDS, JEOL JSM-6510, Tokyo, Japan). The surface functional groups in the biochars were identified via Fourier Transform Infrared Spectroscopy (FTIR, Tensor 27 Bruker Germany) spectrometer using the KBr tablet method in the 4000–400 cm^−1^ wavelength range. The surface mineral compositions were determined via X-ray diffraction (XRD) (D8 Advance, Bruker, Germany).

### 2.3. Batch Sorption Experiments

All sorption experiments used 0.01 mol·L^−1^ NaNO_3_ as the background electrolyte, and a 0.1 mol·L^−1^ HNO_3_ or NaOH solution was used to adjust the initial pH value of the required solution to 5.0 ± 0.05 (except for experiments with different initial pH values). Single-solute sorption experiments were conducted by adding 30 mg of SASC (*w/v*, 1 g·L^−1^) to 30 mL of a Cd(II) or Cu(II) or Zn(II) solution at 25 °C and 200 rpm. The initial pH of the solution was adjusted to 2.0–6.0 to determine the effects of various initial pH values on the sorption of heavy metals by the biochars.

To investigate the sorption kinetics, the initial concentration of the solution was 100 mg·L^−1^, and samples were obtained at various time intervals that ranged between 0.17 and 48 h. To obtain the sorption isotherm, the concentration of the initial solution was 0–250 mg·L^−1^, and it was shaken for 24 h. According to the kinetic experiment, when the adsorption reached 24 h, the adsorption capacity did not change apparently, and the adsorption basically reached equilibrium. After oscillation, the adsorptive solution was collected and filtered with a 0.22-micron-aperture filter. The amount of remnant heavy metal ions in the filtrates was determined via ICP-OES. All adsorption experiments were performed in triplicate, and the average value was taken as the result, while the value for a blank without an added sample was used as a correction to eliminate possible errors. For the FTIR, XRD, SEM, and EDS analyses, 100 mL of a heavy metal solution with a concentration of 1000 mg·L^−1^ was added to 1 g of SASC at pH 5.0 ± 0.05 to prepare a biochar that was loaded with Cu(II), Zn(II), or Cd(II).

### 2.4. Contribution of Each Mechanism to the Biochar Sorption

According to the calculation method of Cui [21], the sorption process of heavy metal ions by the biochar could be attributed to four mechanisms: (1) exchange with cations (Q_ce_), (2) precipitation with minerals (Q_cp_), (3) complexation with oxygen functional groups (OFGs) (Q_co_), and (4) coordination with π electrons (Q_cπ_). Other possible sorption mechanisms were not considered in the experiment due to their low contributions.

(1) The contribution of cation exchange (K^+^, Ca^2+^, Na^+^ and Mg^2+^) depends on the difference in the concentrations of exchangeable cations in the solution before and after biochar sorption, which could be calculated from the difference in the amounts of exchangeable cations that are released between normal sorption and sorption without heavy metals.

(1) $$Q_{\mathrm{ce}}{\;=\;Q}_{K}{\;+\;Q}_{\mathrm{Ca}}{\;+\;Q}_{\mathrm{Na}}{\;+\;Q}_{\mathrm{Mg}}$$

where Q_K_, Q_C__a_, Q_Na_, and Q_Mg_ are the net values of K^+^, Ca^2+^, Na^+^ and Mg^2+^, respectively, in mg·g^−1^ that are released into the solution by the SASC sorption process.

(2) Most minerals in biochar are removed after acid leaching, while the oxygen-containing functional groups (OFGs) are unchanged; hence, the contributions of mineral precipitation (Q_cp_) and ion exchange (Q_ce_) can be calculated from the reduction in biochar sorption before versus after acid leaching.

(2) $$Q_{\mathrm{cp}}\;=\;Q_{t}\;-\;Q_{a}\;\times \;y\;-\;Q_{\mathrm{ce}}$$

where Q_t_ (mg·g^−1^) is the total sorption of SASC, Q_a_ is the amount of sorption on acid-washed biochar, and  y is the yield of acid-washed biochar.

(3) The following chemical reaction formula explains the drop in the solution pH after acid-washed biochar sorption:

−COOH + Cu^2+^ + H_2_O → −COOCu^+^ + H_3_O^+^ (3)

−OH + Cu^2+^ + H_2_O → −OCu^+^ + H_3_O^+^. (4)

Therefore, the contribution of OFGs could be calculated from the drop in the solution pH value.

The sorption capacity of the acid-washed biochar is the result of the interactions between π electrons and OFGs. Therefore, the contribution of π electrons to adsorption could be calculated from the difference between the adsorptions of the acid-washed biochar and OFGs.

(5) $$Q_{c\pi }\;=\;Q_{a}\;\times \;y\;-\;Q_{\mathrm{co}}$$

The contribution rates of these mechanisms in the sorption process are expressed as Q_cp_/Q_t_, Q_co_/Qt, Q_ce_/Q_t_, Q_c__π_/Q_t_.

## 3. Results

### 3.1. Characteristics of Biochar

The composition of the feedstock has a decisive influence on the characteristics of the biochar. Compared with other biochar feedstocks, spent *Agaricus bisporus* substrates are complex mixtures that are derived from edible fungal cultivation. In the process of cultivation, straw, chicken manure, and gypsum are used as nutrients, and peat soil is applied as a cover to provide a growth environment. The yield and ash content of biochars from spent *Agaricus bisporus* substrates exceeded those from common feedstock (e.g., wheat straw [25], halophyte [26], and corn straw [14]).

The high mineral content results in a very high yield of SASC, which is still 64.6% even at the highest pyrolysis temperature, and such a high yield can be attributed to the higher ash content in the biochar. The ash content reached a maximum of 82.1% at 750 °C, which supported the presence of large amounts of inorganic minerals in spent *Agaricus bisporus*-derived biochars.

The pyrolysis temperature is another key factor that affects the properties of a biochar [27]. The properties of SASC are summarized in Table 1. Element analyses showed that the contents of C, H, O, and N decreased continuously with the increase of the pyrolysis temperature. The contents of C, H, N and O are 17.5%, 1.3%, 13.5%, and 1.5%, respectively, after pyrolysis at 350 °C, and they decline rapidly to 12.2%, 0.3%, 4.65%, and 0.85%, respectively, at 750 °C. The ratios H/C and O/C also decrease with the pyrolysis temperature. These results demonstrate that higher-temperature biochar had stronger aromaticity and polarity, which was supported by the subsequent FTIR results. During pyrolysis, many carbon-containing substances will be converted into gaseous hydrocarbon compounds and aromatic hydrocarbons of tar [28], and the gradual loss of volatile substances will remove many surface functional group elements (H, O and N).

Mineral element analyses of ICP showed that SASC included calcium (Ca), magnesium (Mg), potassium (K), and sodium (Na) (Table 1, Table S1), which were controlled mainly by the production temperature. The total content of Ca, Mg, K, and Na increased from 10.41% to 11.39% as the production temperature was increased from 350 to 750 °C. During pyrolysis, these inorganic minerals were not easily volatilized [29] and were retained and enriched in the biochars, while organic substances (such as hemicellulose, lignin and cellulose (220–400 °C) and lignin (~500 °C) [30]) were gradually volatilized and lost, thereby resulting in an increase in the mineral element content in the biochars with increasing temperature.

Minerals are also key factors that affect the acidity or alkalinity of biochars. When not pyrolyzed, the pH of the raw material of SASC was 6.87, which was slightly acidic. As the pyrolysis temperature was increased from 350 to 750 °C, the pH of the biochar increased from 8.83 to 11.82. The alkaline earth metals (Ca and Mg) in biochars are converted into carbonate forms during pyrolysis [31] (such as CaMg(CO_3_)_2_ and CaCO_3_), which render the pH alkaline and gradually increase it. Meanwhile, SASC had a higher Ca content (5.13~7.77%); hence, more alkaline minerals can be released to cause the alkaline elevation of SASC.

The specific surface area and pore volume of the biochars varied substantially with the pyrolysis temperature. Due to the formation of micropore structures in the biochars during pyrolysis, the specific surface area and porosity of the biochars increased significantly with the pyrolysis temperature [32]. As the temperature was increased from 350 to 650 °C, the total pore volume of the biochars increased from 0.045 to 0.156 cm^3^·g^−1^, and the surface area increased from 36.20 to 101.39 m^2^·g^−1^. However, when the temperature was 750 °C, the pore volume and surface area decreased to 0.091 and 37.08 m^2^·g^−1^, respectively. The surface area of SAS750 is lower than that of the common straw biochar [33,34]. The pores of the biochar were blocked by excessive ash [35], which decreased the specific surface area and pore volume. In addition, the pore structure of the biochar collapsed at high pyrolysis temperatures [36] (>700 °C), which further reduced the surface area of SAS750. SEM images (Figure S1) show that the surface of the biochar was rough and contained complex networks and porous structures, which further supported the lower specific surface area of SASC. These structures became more complex and disordered as the pyrolysis temperature was increased.

### 3.2. Effect of the Initial pH on the Sorption Performance

The pH of the solution is an important factor that affects the sorption performance. It affects the forms of the ions and the protonation or deprotonation state of the biochar [37]. To explore the influence of the pH on the sorption of Cu(II), Zn(II), and Cd(II) by SASC, a range of initial pH values from 2.0 to 6.0 was selected for sorption experiments.

**Figure 1:**
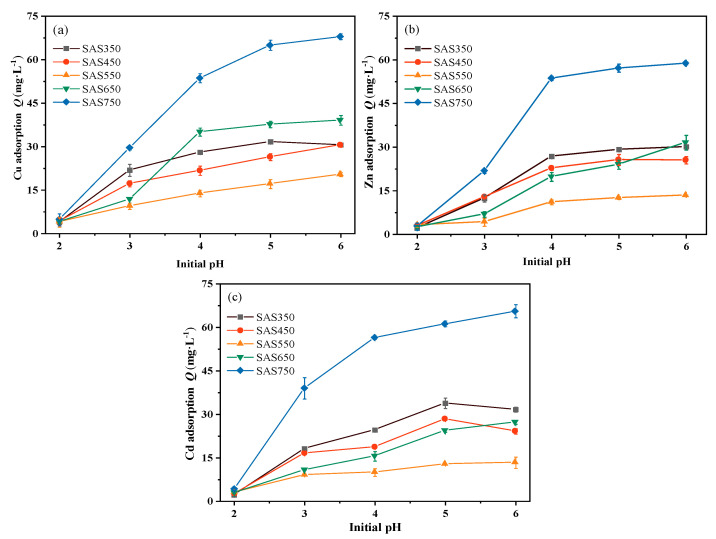
The effects of different solution pH on the sorption capacity of Cu(II) (**a**), Zn(II) (**b**), and Cd(II) (**c**), respectively.

The results are presented in Figure 1. The sorption capacity of each biochar for heavy metals increased gradually with the initial pH value of the solution. When the pH value of the solution was low (pH = 2.0), a large amount of H^+^ was present in the solution, and the biochar was protonated and electrostatically repulsed with positively charged heavy metal ions. Simultaneously, the biochar released many cations (such as Ca^2+^, Mg^2+^, and K^+^) in the low pH solution (e.g., Zn(II) adsorption, Figure S2), which competed with heavy metals for sorption sites and, thus, reduced the adsorption capacity [38]. As the pH value was increased to 3.0, the H^+^ content in the solution decreased, and the biochar was deprotonated. The unfavorable conditions that are described above for biochar sorption weakened; consequently, the adsorption capacity substantially increased.

**Figure 2:**
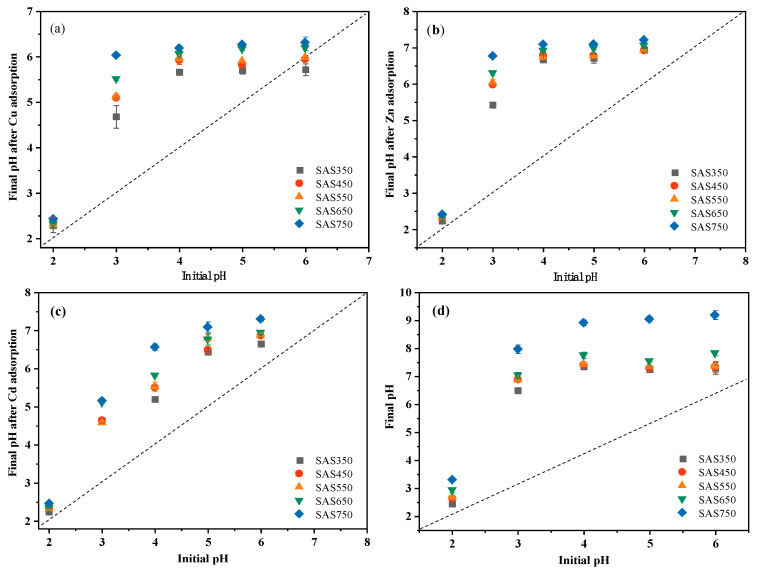
The pH changes of Cu(II) (**a**), Zn(II) (**b**), and Cd(II) (**c**) solutions after adsorption equilibrium, respectively. (**d**) Change of pH of solution without heavy metals after equilibrium.

After sorption, the pH value of the solution showed an upward trend compared with the initial value, which was attributed to the alkalinity of the biochar: the stronger the alkalinity of the biochar, the larger the pH increase (Figure 2a–c). However, for a blank without heavy metals (with the same volume of NaNO_3_ solution), the pH value of the solution after shaking exceeded that of the solution with heavy metals. Thus, the interaction of biochar with heavy metals reduced the pH value of the solution, which is consistent with the results of Wang et al. [39]. When metal ions are complexed with functional groups in the biochar, H^+^ will be released in the solution, which will reduce the pH value of the solution after sorption; this will also be reflected in the sorption performance of the acid-washed biochar. In addition, the decrease in the pH value after sorption may also be due to the formation of precipitates with alkaline ions (CO_3_^2−^) during the sorption process [21]. With increasing pyrolysis temperature, the ash (inorganic component) content of the biochar increased gradually; however, the amounts of adsorbed Cu(II), Cd(II) and Zn(II) initially decreased and subsequently increased (SAS750 > SAS350 > SAS550) (Figure 2e).

Compared with SAS550, SAS750 has a higher pH, ash content, mineral content, and aromaticity and is more suitable for precipitation and cation exchange, while SAS350 contains more abundant oxygen-containing functional groups and is more conducive to oxygen functional group complexation. Therefore, SAS350, SAS550, and SAS750 are selected as representatives for the next kinetic and isothermal sorption experiments.

### 3.3. Sorption Kinetics and Isotherms

**Figure 3:**
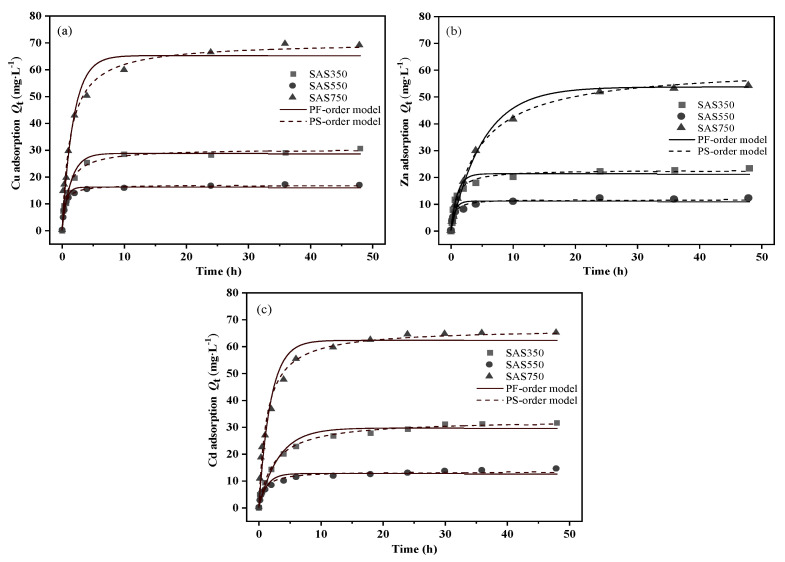
Kinetics of sorption of Cu(II) (**a**), Zn(II) (**b**), and Cd(II) (**c**) on biochars at 350, 550, and 750 °C.

The results of the sorption amounts of the SASCs on Cu(II), Zn(II), and Cd(II) as functions of the sorption time are presented in Figure 3. With increasing sorption time, the sorption capacities of the SASCs gradually increased until equilibrium. At the initial sorption time (0–4 h), all three SASCs showed rapid sorption. As the sorption time progressed, the solute difference of the solution decreased, the remaining sorption sites on the SASCs gradually became saturated, with little change in the sorption capacity, and the sorption reached equilibrium. Although SAS750 exhibited the best sorption performance for each heavy metal, it exhibited a lower sorption rate and did not reach equilibrium until nearly 24 h, which may be attributed to differences in the sorption mechanisms. The sorption rates differ among the sorption mechanisms. According to the result of LU H [40], the interactions between oxygen-containing functional groups and heavy metal ions in biochars are extremely rapid, and equilibrium can usually be reached in a short time. During the sorption process, anions that are released from biochars (e.g., CO_3_^2−^, SO_4_^2−^, and OH^−^) can precipitate with heavy metals, while the rate of mineral precipitation is affected by the release of anions [41]. Biochars under high-temperature pyrolysis have few oxygen-containing functional groups and high mineral content, which is more conducive to the removal of heavy metals through precipitation mechanisms; thus, the sorption rate is strongly affected by minerals.

Minerals in biochars at low temperatures are amorphous and more easily released to bind to heavy metals [42]. Increasing the pyrolysis temperature (>550 °C) causes the minerals in biochars to become more crystalline, which decreases the release rate and limits the corresponding sorption rate. SAS750 requires the longest time to reach equilibrium, which may be due to the lower contribution of functional groups and the higher contribution of minerals in the sorption process (Figure S4).

Pseudo-first-order [43] and pseudo-second-order [44] kinetic models were used to explore the adsorption process of biochars (Figure 3 and Table S2). According to the fitted regression coefficient (R_2_), the pseudo-second-order model of the sorption processes of SASCs with various pyrolysis temperatures better described the sorption of Cu(II), Zn(II), and Cd(II) than the pseudo-first-order. The pseudo-second-order model assumes that the rate-limiting step involves chemical interactions leading to the binding of the ions to the surface by strong covalent bonding [45]. As the reaction kinetics-based models are applicable in the adsorption process, the basic assumption of these models is that the mass transfer is fast enough to be ignored. Accordingly, these models are applied on the chemisorption of solids that are porous, exhibiting high solid-phase diffusion coefficients, in this way, that are appropriate for biochar [45].

**Figure 4:**
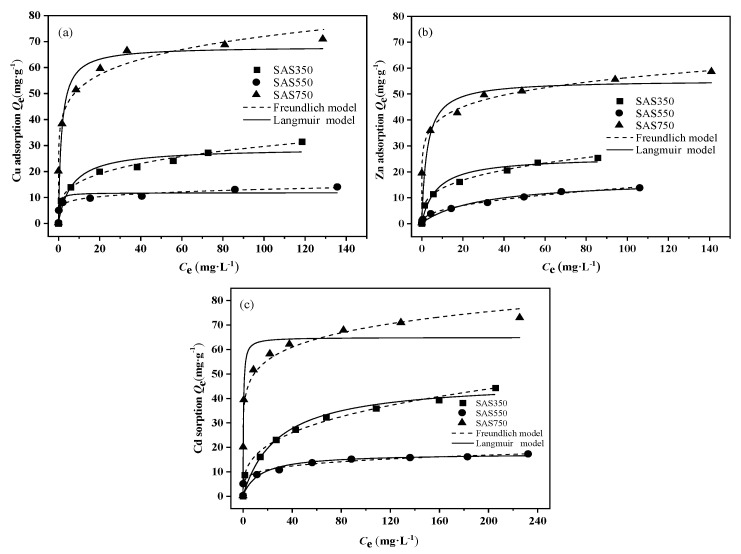
Isotherm model of sorption of Cu(II) (**a**), Zn(II), (**b**) and Cd(II) (**c**) on biochars at 350, 550, and 750 °C.

The Langmuir and Freundlich models were used to fit the sorption data. The fitting results are presented in Figure 4 and Table S3. The fitting results of the Freundlich model better describe the equilibrium data than those of the Langmuir model; thus, the sorption of SASC was heterogeneous adsorption [46]. All n values exceeded 1.0; hence, SASC had substantial heterogeneity in sorption affinity for Cu(II), Zn(II), and Cd(II). The Langmuir maximum sorption capacity (Q_max_) followed the order SAS750 > SAS350 > SAS550, and the sorption capacity of SAS750 exceeded those of biochars from other feedstocks that were reported in many studies (Table S3). Overall, for Cu(II) sorption: 68.1 mg·g^−1^ > 28.9 mg·g^−1^ > 11.6 mg·g^−1^; for Zn(II) sorption: 55.2 mg·g^−1^ > 25.6 mg·g^−1^ > 16.9 mg·g^−1^; and for Cd(II) sorption: 64.8 mg·g^−1^ > 47.2 mg·g^−1^ > 17.2 mg·g^−1^. These results demonstrate that SAS750 has a higher removal capacity for Cu(II), Zn(II), and Cd(II).

### 3.4. Sorption Mechanism Analysis

To investigate the sorption mechanisms of Cu(II), Zn(II), and Cd(II) on SASC, samples were scanned via FTIR, XRD, and SEM/EDS before and after sorption.

#### 3.4.1. Metal Cation Exchange

**Figure 5:**
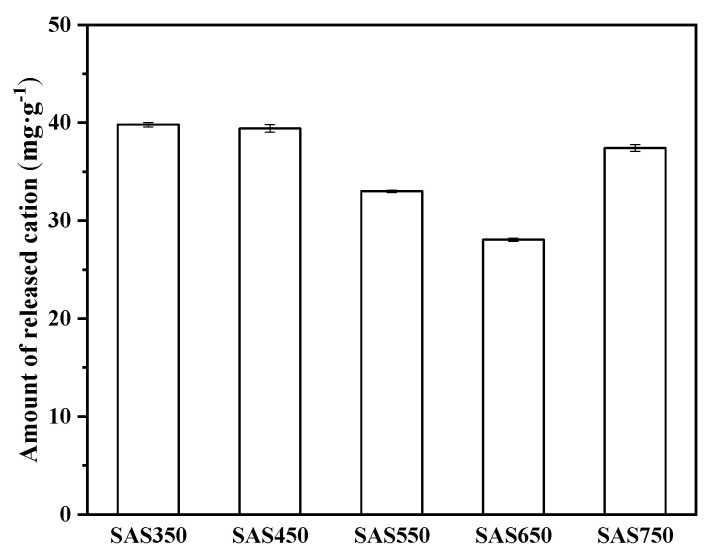
The amount of total cation released from spent *Agaricus bisporus* substrate biochars (SABCs) into solution (initial pH of 5, 24 h, adsorbent dosage of 1 g·L^−1^).

**Figure 6:**
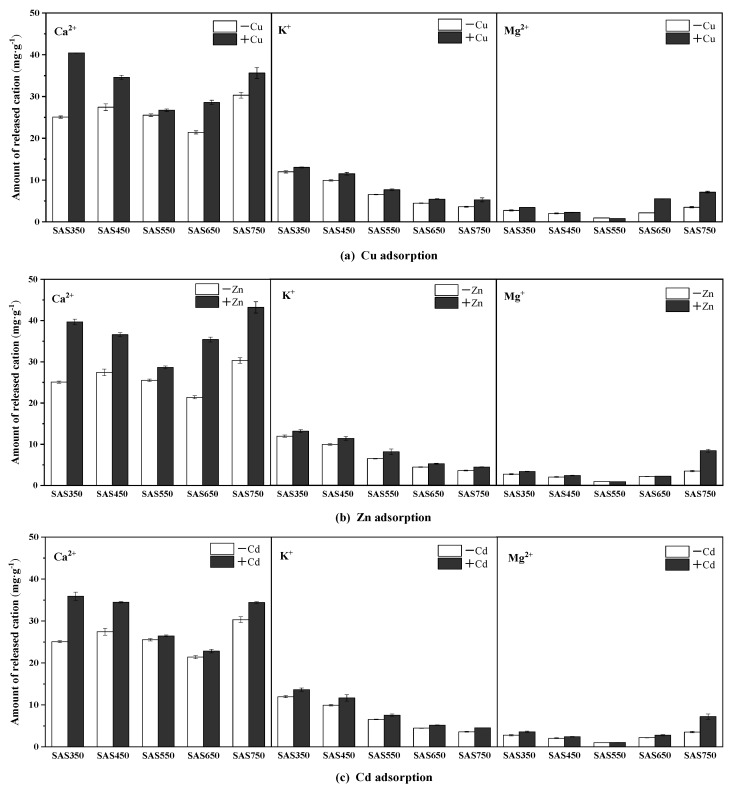
The amount of Ca^2+^, K^+^, and Mg^2+^ released from spent *Agaricus bisporus* substrate biochars (SABCs) into solution before and after Cu(II) (**a**), Zn(II) (**b**), and Cd(II) (**c**) adsorption (pH of 5, 24 h, initial concentration of 100 mg·L^−1^ and adsorbent dosage of 1 g·L^−1^).

Cations on the surface of biochars (e.g., Ca^2+^, K^+^, Mg^2+^, and Na^+^) can exchange with heavy metal ions in solution. To investigate this phenomenon, the release of these cations into solutions that contained and did not contain heavy metals was measured. The results are presented in Figure 5 and Figure 6. In the blank experiment (without heavy metals), many cations were released from the biochar into the solution, especially Ca^2+^. When the pyrolysis temperature was increased, the total amount of cations that were released from the biochar gradually decreased and reached the lowest value at 650 °C, whereas when the temperature increased to 750 °C, the amount of cations that were released from the biochar increased (Figure 5).

Compared with the blank experiment, the released amounts of Ca^2+^ and K^+^ in the solution after SASC sorption (containing heavy metals) increased, while the released amount of Mg^2+^ changed substantially only in SAS750. (Figure 6) In most cases [47], the cation exchange during the sorption of low-temperature biochar (~550 °C) was stronger than that at high-temperature (≈750 °C). In this study, it was found that the increment of cation release from the solution after the pyrolyzed biochar adsorbed heavy metals at 550 °C was apparently lower than those at other pyrolysis temperatures. For example, for the sorption of Cu(II), the samples are ordered according to the total amount of cations that were released as follows: SAS550 (3.23 mg·g^−1^) < SAS650 < (9.49 mg·g^−1^) < SAS450 (9.61 mg·g^−1^) SAS750 < (11.59 mg·g^−1^) < SAS350 (18.09 mg·g^−1^). The reasons for this could be mainly attributed to the structure of crystalline minerals at 550 °C [21]. These results demonstrate that the pyrolysis temperature affects the process of cation exchange in the sorption of heavy metals by spent mushroom substrate biochar.

#### 3.4.2. Precipitation with Minerals

**Figure 7:**
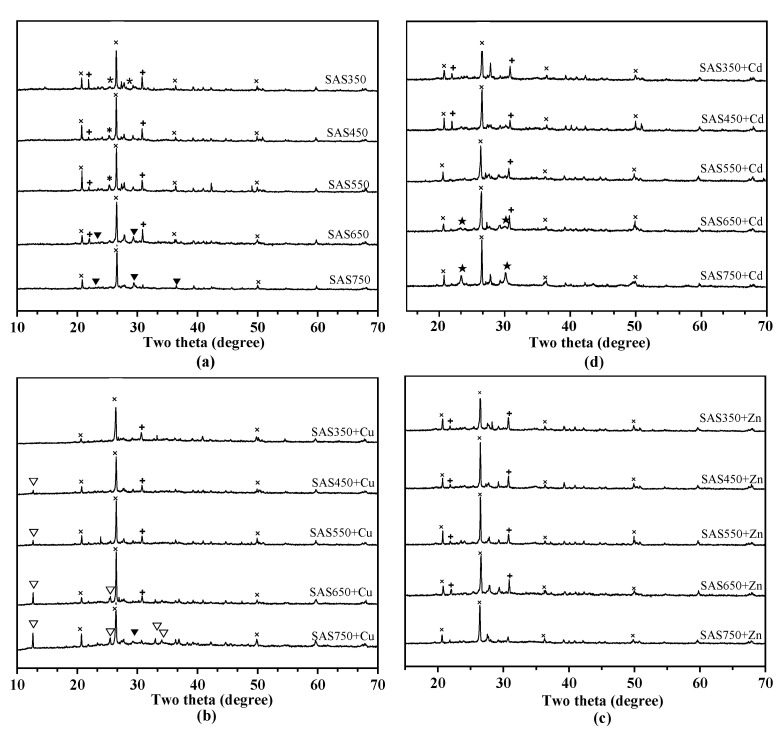
X-ray diffraction (XRD) patterns of biochars before (**a**) and after adsorbed with metal of Cu(II) (**b**), Zn(II) (**c**), or Cd(II) (**d**). Minerals with peaks labeled ×, quartz (SiO_2_); *, calcite (CaSO_4_); ▼, calcium carbonate (CaCO_3_); ✚, dolomite (CaMg(CO_3_)_2_); ▽, posnjakite (Cu_4_SO_4_(OH)_6_H_2_O); ★, Otavite (CdCO_3_).

Anions that are released from biochar (e.g., OH^−^, CO_3_^2−^, PO_4_^3−^, and SO_4_^2−^) can precipitate with heavy metal ions. According to a previous prediction, mineral precipitation will play an important role in the sorption process of SASCs, especially for biochars under high-temperature pyrolysis. To evaluate the role of precipitation in the sorption process, biochars before and after sorption were scanned using XRD (Figure 7a). The calcium-containing minerals that were added into the culture medium of Agaricus bisporus as the calcium source were not completely absorbed by Agaricus bisporus, and calcium sulfate (CaSO_4_), dolomite (CaMg(CO_3_)_2_), and calcite (CaCO_3_) were detected after pyrolysis via XRD (Figure 7a). With increasing temperature, CaSO_4_ peaks disappeared, while CaCO_3_ peaks were newly formed in SAS650 and SAS750. Meanwhile, the peak of CaMg(CO_3_)_2_ weakened until it disappeared at 750 °C. Consistent with the results of the EDS spectra (Figure S1), there were strong peaks that were attributed to SiO_2_ in the XRD patterns of each biochar; hence, SiO_2_ was abundant in SASC.

Compared with unabsorbed samples, new peaks that represented precipitates were observed for the samples after Cd(II) and Cu(II) sorption, especially for biochars under high-temperature pyrolysis (>550 °C). After the sorption of Cu(II), posnjakite (Cu_4_(SO_4_)(OH)_6_(H_2_O)) was formed (Figure 7b), and the peak intensity increased with the pyrolysis temperature. After the sorption of Cd(II), new peaks of Otavite (CdCO_3_) were formed in SAS650 and SAS750 (Figure 7d). No precipitate was readily identified in the XRD spectra after the sorption of Zn(II). This result suggests that no significant precipitation occurred during the sorption of Zn(II). Compared with other samples, SAS750 showed the highest sorption capacity for each heavy metal; hence, it was chosen for the SEM and EDS analyses (Figure S2). Compared with nonadsorbed biochars, many precipitates were observed in the SEM images of SAB750 after heavy metal adsorption, which were flocculent for Cd and flaky for Cu and Zn. The EDS spectra further showed the elemental composition (Figure S2), and it was found that the presence of Cd, Cu, and Zn elements and the proportion of Ca elements in the samples decreased significantly after sorption. These results supported the important roles of mineral precipitation and cation exchange in the sorption process of high-temperature biochars.

#### 3.4.3. Oxygen Functional Group and π Electrons

**Figure 8:**
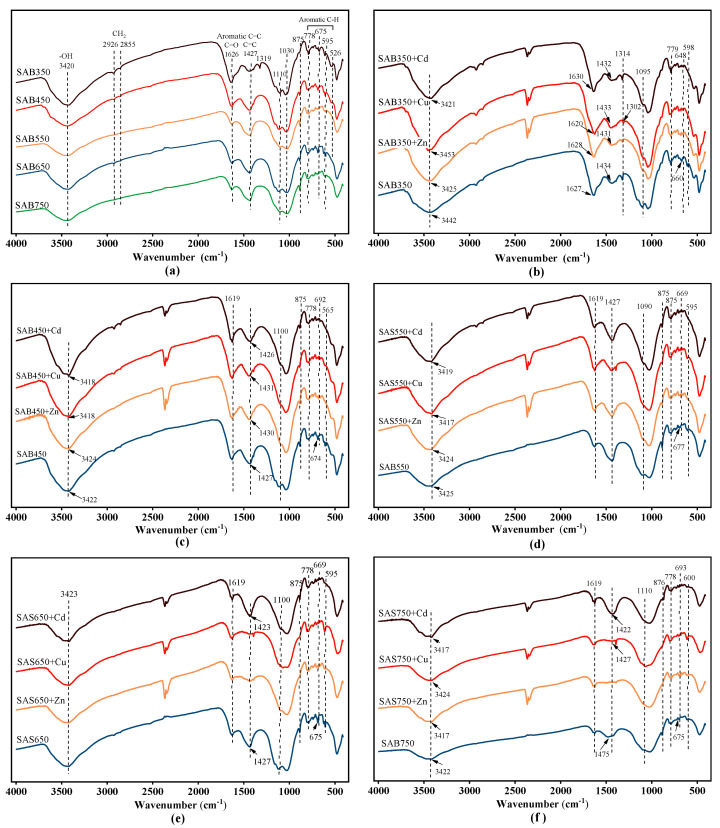
(**a**) Fourier transform infrared spectra (FTIR) spectra of biochar at 350–750 °C pyrolysis temperature. FTIR spectra of SAS350 (**b**), SAS450 (**c**), SAS550 (**d**), SAS650 (**e**), and SAS750 (**f**) before and after sorption.

FTIR spectra before and after sorption are shown in Figure 8. Functional groups (e.g., C=C, -COOH, -OH, and R-OH) in biochar have an important influence on the sorption process of heavy metals [48]. The typical bands at 3420 cm^−1^ are attributed to -OH vibrations, the bands at 2950–2850 cm^−1^ are attributed to aliphatic C-H stretching [49,50], the bands at 1620 cm^−1^ are attributed to C=O vibrations of carboxyl groups, and the bands at 1319 cm^−1^ are attributed to C-O peaks. C=C skeleton vibration of the aromatic ring corresponds to the band at 1427 cm^−1^, and C-H bending vibration of the aromatic ring corresponds to the band at 800–600 cm^−1^ (778, 675, 595 cm^−1^) [51]. The band at 1110 cm^−1^ can be attributed to SO_4_^2−^.

After the pyrolysis temperature was increased, the vibration of the corresponding -OH gradually weakened, the aliphatic C-H stretching weakened and disappeared, the C-O stretching disappeared at temperatures above 450 °C, and the C=O vibration of the carboxyl group continued to weaken. In addition, the SO_4_^2−^ stretching continuously weakened as the temperature was increased to 550 °C. The corresponding vibration of aromatics was enhanced with increasing temperature, and the enhancement of the aromaticity can provide more π electrons to bind with heavy metals. In addition, the related vibrations at 875 cm^−1^ were assigned to CO_3_^2−^ [22], and the vibrations of Si-O-Si at 465 cm^−1^ and 1030 cm^−1^ were assigned to SiO_2_, which was consistent with the XRD analysis results.

After sorption, each functional group changed, and the position of the corresponding peak shifted (Figure 8b–f). For example, the peaks at 1620, 1319, and 1100 cm^−1^ (C=O, C-O, and C-O-C vibrations, respectively) were weakened, while the changes in the oxygen functional groups of low-temperature biochars were more obvious; thus, more oxygen functional groups were involved in the sorption of low-temperature pyrolysis biochars. In addition, the pH value of the solution decreased after the sorption of acid-washed biochar, which also supported the involvement of the oxygen functional groups in the sorption process.

In addition to oxygen functional groups, other functional group components (aromatic C=C and C-H) have also been demonstrated to be involved in the sorption process, especially the interactions of π with Cu(II), Zn(II), and Cd(II). It is observed that the C-H vibration of the peak at 800~600 cm^−1^ continues to weaken or be displaced. Especially for Cu(II) and Zn(II) sorption, the C=C change at 1427 cm^−1^ at high temperatures (≥650 °C) is more drastic, which is attributed to the high degree of graphitization at high pyrolysis temperatures. At high temperatures (≥650 °C), the change that corresponds to the CO_3_^2−^ peak at 875 cm^−1^ became more obvious; thus, more CO_3_^2−^ participated in the precipitation reaction at high temperatures and less participated at low temperatures.

### 3.5. Contributions of the Cu(II), Zn(II), and Cd(II) Sorption Mechanisms

**Figure 9:**
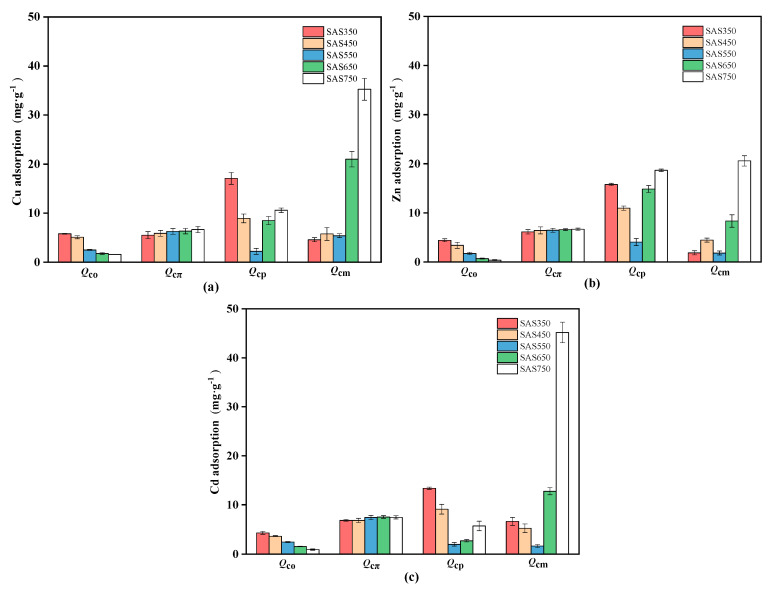
The change of contributions of different adsorption mechanisms with temperature during Cu(II) (**a**), Zn(II) (**b**), and Cd(II) (**c**) adsorption (pH of 5, 24 h, initial concentration of 100 mg·L^−1^ and adsorbent dosage of 1 g·L^−1^).

The contributions of various mechanisms to the process of heavy metal sorption by biochars were evaluated according to the method that is described in the Materials and Methods section. The contribution amounts and contribution proportions of various sorption mechanisms are presented in Figure 9 and Table S4. In the sorption of the three considered heavy metals, the contribution of π coordination (Q_cπ_) gradually increased as the pyrolysis temperature was increased (350–750 °C), while the contribution of oxygen-containing functional group complexation (Q_co_) showed the opposite trend. For instance, in the process of SASC adsorption of Cu(II), Q_cπ_ increased from 5.52 to 6.66 mg·g^−1^ with increasing temperature (350–750 °C), while Q_co_ and Q_co_/Q_t_ decreased from 5.81 mg·g^−1^ and 18.4% to 1.62 mg·g^−1^ and 2.0%, respectively. This phenomenon may be due to the reserved amount of oxygen functional groups and the strength of the aromaticity, which are affected by the pyrolysis temperature. The sorption of Cu(II) and Zn(II) by different mechanisms showed similar trends.

The variation rules of Q_co_ and Q_cπ_ are strongly influenced by the amount of oxygen-containing functional groups and the aromaticity in biochars. FTIR analysis showed that pyrolysis enhanced the aromaticity and decomposition of oxygen-containing functional groups in SASCs, thereby resulting in a gradual decrease in the contribution of oxygen functional groups to sorption, while the role of π electrons was constantly enhanced.

The change in the cation exchange contribution (Q_ce_) with temperature was substantial in several sorption mechanisms. When the pyrolysis temperature was between 350 and 550 °C, the cation exchange (Q_ce_) decreased with the increasing pyrolysis temperature, whereas when the pyrolysis temperature was increased from 550 to 750 °C, Q_cp_ showed the opposite upward trend. The values of Q_ce_ for Cu(II), Zn(II), and Cd(II) sorption were only 2.23, 1.83, and 4.05 mg·g^−1^, respectively, for SAS550. The abrupt decrease in Q_ce_ in SAS550 resulted in the weakest sorption performance. Lower pyrolysis temperatures were conducive to the sorption of heavy metals via cation exchange mechanisms, at which time biochar had a lower degree of carbonization and could release many available mineral components (such as Ca^+^ and K^+^). When the pyrolysis temperature was increased from 350 to 550 °C, the mineral components in SASC became more crystalline [52], and the solubility decreased, thereby resulting in a decrease in the cation exchange capacity. When the pyrolysis temperature was further increased (>650 °C), the cation exchange capacity did not continue to decline but began to increase. At this pyrolysis temperature, high-temperature pyrolysis resulted in a change in the mineral crystals in SASC with the production of new minerals [41] (Figure 7a; CaMg(CO_3_)_2_ is converted to CaCO_3_), thereby resulting in the solubility of minerals in the biochar being no longer reduced, which was manifested as enhanced exchange of Ca^2+^ and Mg^2+^ cations with heavy metal ions (Table S3)

Compared with the high pyrolysis temperature, a small amount of precipitation occurred during the sorption process of the biochar at low temperatures (350–550 °C). The XRD pattern showed a weak representative precipitate peak (for Cu(II)) or no readily observable precipitate (for Cd(II) and Zn(II)) (Figure 7c,d), especially for SAS350 after sorption. The mineral precipitation ratios in the sorption of the high-pyrolysis-temperature biochars (SAS650 and SAS750) increased (as shown in Figure 7c,d); this change was also demonstrated by the XRD pattern.

Overall, the pyrolysis temperature affects the ability of biochars to adsorb heavy metals, and the dominant mechanism of biochar adsorption of heavy metals differs among pyrolysis temperatures. For the organic components (Q_cπ_ + Q_co_), the contribution gradually decreased with increasing temperature, while the inorganic components (Q_ce_ + Q_cp_) were more substantially affected by the pyrolysis temperature, and the contribution initially decreased and subsequently increased. The optimal production temperature is 750 °C for pyrolysis. At this temperature, the contribution of the mineral precipitation increased substantially, the sorption amount reached its maximum value, and the Q_cp_/Q_t_ values for Cu(II), Zn(II), and Cd(II) were 63.4%, 44.4%, and 71.2%, respectively.

## 4. Conclusions

Mineral-rich biochar that was derived from a spent *Agaricus bisporus* substrate showed the effective removal of Cu(II), Zn(II), and Cd(II) from aqueous solutions, and the sorption performance was affected by the pyrolysis temperature and the solution pH value. The pyrolysis temperature of 750 °C yields the best adsorbent. According to mechanistic investigations, mineral components play a key role in biochar sorption, which is mainly through cation exchange at low pyrolysis temperatures (e.g., 350 °C), whereas mineral precipitation plays a major role at high temperatures (e.g., 750 °C). In conclusion, the results suggested that the production of a mineral-rich biochar from a spent *Agaricus bisporus* substrate for the removal of heavy metals from aqueous solutions is a promising method for the utilization of abandoned spent mushroom substrates.

## Figures and Tables

**Table 1:** Physicochemical properties of biochars at different pyrolysis temperature.

Material	Yield (%)	pH	Ash (%)	Elemental Contents (%)								H/C	O/C	BET (m^2^ g^−1^)	Total Pore Volume (cm^3^ g^−1^)
				C	H	O	N	Ca	Mg	K	Na				
SAS	-	6.87	53.15	19.03	2.33	23.82	1.67	5.13	1.48	0.60	0.35	0.12	1.25	24.13	0.029
SAS350	78.96	8.83	66.50	17.53	1.32	13.49	1.48	6.45	1.84	0.64	0.39	0.08	0.77	36.20	0.045
SAS450	73.73	9.37	71.76	15.91	0.82	11.73	1.20	7.13	1.97	0.68	0.42	0.05	0.78	48.74	0.065
SAS550	70.98	9.51	74.08	15.22	0.54	9.87	1.06	7.30	2.11	0.69	0.43	0.04	0.65	64.19	0.110
SAS650	68.13	11.38	77.23	14.53	0.37	7.87	0.88	7.45	2.21	0.70	0.44	0.03	0.54	101.39	0.156
SAS750	64.60	11.82	82.12	12.17	0.26	4.65	0.85	7.77	2.41	0.76	0.45	0.02	0.38	37.08	0.091

## Data Availability

The data presented in this study are available in supplementary material.

## Supplementary Materials

The following are available online at https://www.mdpi.com/1996-1944/14/1/35/s1; Mathematical models for adsorption kinetics and isotherm; Table S1: The concentrations of metals in biochars derived from spent Agaricus bisporus substrate; Table S2: Pseudo-first (PF) order and pseudo-second (PS) order model parameters for the Cd(II), Cu(II), or Zn(II) sorption onto SAS-derived biochars produced at 350, 550, and 750 °C.; Table S3: Langmuir and Freundlich isotherm parameters for Cd(II), Cu(II), or Zn(II) sorption onto SAS-derived biochars produced at 350, 550, and 750 °C; Table S4: Comparison of SASC adsorption capacity of Cd(II), Cu(II), and Zn(II) with other biochar; Figure S1: The SEM images (3000×) and corresponding EDS spectra of spent Agaricus bisporus derived biochars at 350–750 °C; Figure S2: The SEM images (3000×) and corresponding EDS spectra of spent Agaricus bisporus derived biochars after adsorption of Cd(II), Cu(II), and Zn(II) at 750 °C; Figure S3: The amount of Ca2+, K+ and Mg2+ released from SABCs into solution after Zn(II) adsorption at different initial pH values; Figure S4: The contribution percentage of different mechanisms to Cu(II) (a), Zn(II) (b), and Cd(II) (c) sorption on SASCs.
