# Supplementary material for: Efficient Removal of Cu(II), Zn(II), and Cd(II) from Aqueous Solutions by a Mineral-Rich Biochar Derived from a Spent Mushroom (Agaricus bisporus) Substrate
Source: Materials (Basel). 2020 Dec 23;14(1):35. doi: 10.3390/ma14010035 (PMC7794708; doi:10.3390/ma14010035)
Supplement: Supplementary file 1 [file materials-14-00035-s001.pdf]

materials

Supplementary Materials

Efficient Removal of Cu(II), Zn(II), and Cd(II) from Aqueous Solutions by a Mineral-Rich Biochar Derived from a Spent Mushroom (*Agaricus bisporus*) Substrate

Guosheng Zhang [1], Na Liu [1], Yuan Luo [1], Haibo Zhang [1], Long Su [1], Kokyo OH [2] and Hongyan Cheng [1,*]

[1] College of Resources and Environment, Shanxi Agricultural University, Taigu 030801, China; zhanggs@stu.sxau.edu.cn (G.Z.); liuna@sxau.edu.cn (N.L.); luoyuan@sxau.edu.cn (Y.L.); zhanghb@sxau.edu.cn (H.Z.); sulong@stu.sxau.edu.cn (L.S.)

[2] Center for Environmental Science in Saitama, Kazo City, Saitama 347-0115, Japan; peace6728@yahoo.co.jp

* Correspondence: sxauchenghy@sxau.edu.cn

Supplementary Materials and Method

Mathematical Models for Adsorption Kinetics and Isotherm

The adsorption capacity of spent Agaricus bisporus substrate biochar was calculated according to Equation (1):

$$Q_e=(C_0-C_e)\cdot\frac{V}{m} \tag{1}$$

where Qe ($mg\cdot g^{-1}$) is the adsorption capacity of biochar at equilibrium; V (L) is the solution volume; C0 ($mg\cdot L^{-1}$) is the initial heavy metal (Cd(II) or Cu(II) or Zn(II)) concentration; C_e is the Cd(II) or Cu(II) or Zn(II) concentration after time adsorption, and m (g) is the weight of biochar samples.

The kinetics of Cd(II) or Cu(II) or Zn(II) sorption were fitted to the pseudo-first order (PF-order model, Equation (2)), pseudo-second order (PS-order model, Equation (3))

$$Q_t=Q_e(1-e^{-k_1 t}) \tag{2}$$

$$Q_t=\frac{k_2 Q_e^{2} t}{1+k_2 Q_e t} \tag{3}$$

where Qt ($mg\cdot g^{-1}$) is the sorbed amount at time t; k1 (h^{-1}) and k2 ($g\cdot mg^{-1}\cdot h^{-1}$) are the apparent first-order and second-order sorption rate constants, respectively.

The Freundlich isotherm model (Equation (4)) and Langmuir isotherm model (Equation (5)) was used to simulate Cd or Cu or Zn sorption to biochars,

$$Q_e=k_F C_e^{\,n} \tag{4}$$

$$Q_e=\frac{Q_{max} k_L C_e}{1+k_L C_e} \tag{5}$$

Qmax ($mg\cdot g^{-1}$) is the maximum adsorption capacity; KL ($L\cdot mg^{-1}$) is the Langmuir constant related to the adsorption affinity; Kf ($mg\cdot g^{-1}$) is

the Freundlich constant related to the adsorption capacity; n is the adsorption strength.

Table S1. The concentrations of metals in biochars derived from spent *Agaricus Bisporus* substrate.

Biochar	Fe	Mn	Al	Zn (mg·g^{-1})	Cu	Cd	Pb
SAS350	3.233	0.167	14.60	0.165	0.042	N.D	0.014
SAS450	3.173	0.165	15.91	0.146	0.039	N.D	0.009
SAS550	2.997	0.159	16.13	0.145	0.037	N.D	0.007
SAS650	3.270	0.163	16.49	0.140	0.036	N.D	0.004
SAS750	3.434	0.163	17.32	0.135	0.034	N.D	0.003

SAS350-SAS750 were the biochars derived from spent Agaricus bisporus substrate (SAS) at 350–750 °C. N.D.: Not detected (below the detection limit).

Table S2. Pseudo-first (PF) order and pseudo-second (PS) order model parameters for the Cu(II) or Zn(II) or Cd(II) sorption onto SAS-derived biochars produced at 350, 550, and 750 °C.

	Biochars	PF Order Model			PS Order Model		
		Qe(mg·g^{-1})	k_1 (1 h^{-1})	R^2	*Qe* (*mg·g^{-1}*)	k_2 (g mg^{-1} h^{-1})	R^2
**Cu**	SAS350	28.6 ± 0.98	0.691 ± 0.098	0.963	30.4 ± 0.84	0.033 ± 0.005	0.980
	SAS550	16.0 ± 0.38	1.61 ± 0.164	0.974	16.9 ± 0.11	0.141 ± 0.006	0.998
	SAS750	65.2 ± 2.54	0.547 ± 0.080	0.956	70.3 ± 1.82	0.010 ± 0.001	0.985
**Zn**	SAS350	21.2 ± 0.81	0.987 ± 0.149	0.951	22.7 ± 0.46	0.059 ± 0.006	0.988
	SAS550	10.9 ± 0.53	1.55 ± 0.355	0.911	11.7 ± 0.34	0.171 ± 0.030	0.973
	SAS750	52.4 ± 1.20	0.208 ± 0.017	0.990	59.2 ± 0.70	0.004 ± 0.001	0.998
**Cd**	SAS350	29.6 ± 0.81	0.308 ± 0.036	0.972	32.7 ± 0.60	0.013 ± 0.001	0.992
	SAS550	12.9 ± 0.49	0.765 ± 0.147	0.913	13.47 ± 0.35	0.080 ± 0.013	0.971
	SAS750	62.3 ± 1.89	0.524 ± 0.074	0.956	66.72 ± 1.23	0.012 ± 0.001	0.988

Table S3. Langmuir and Freundlich isotherm parameters for Cu(II) or Zn(II) or Cd(II) sorption onto SAS-derived biochars produced at 350, 550, and 750 °C.

	Biochars	Langmuir Model			Freundlich Model		
		Qe(mg·g^{-1})	K_L (mg L^{-1})	R^2	K_F (mg^{1-n} g^{-1} L^{-n})	*n*	R^2
**Cu**	SAS350	28.9 ± 2.11	0.150 ± 0.058	0.927	8.27 ± 0.46	3.623 ± 0.181	0.995
	SAS550	11.6 ± 0.92	2.089 ± 1.39	0.860	6.37 ± 0.37	6.509 ± 0.618	0.986
	SAS750	68.1 ± 4.85	0.640 ± 0.339	0.890	37.3 ± 1.73	7.001 ± 0.601	0.987
**Zn**	SAS350	25.6 ± 1.82	0.138 ± 0.045	0.954	6.64 ± 0.27	3.276 ± 0.114	0.998
	SAS550	16.9 ± 1.79	0.034 ± 0.010	0.970	1.82 ± 0.14	2.293 ± 0.100	0.996
	SAS750	55.2±4.90	0.389 ± 0.274	0.838	29.6 ± 0.65	7.180 ± 0.288	0.998
**Cd**	SAS350	47.2 ± 3.12	0.035 ± 0.008	0.963	7.66 ± 0.58	3.044 ± 0.152	0.993
	SAS550	17.2 ± 1.36	0.072 ± 0.031	0.896	6.02 ± 0.46	5.289 ± 0.459	0.985
	SAS750	64.8 ± 2.82	2.59 ± 0.97	0.930	37.20 ± 2.07	7.501 ± 0.750	0.977

Table S4. Comparison of SASC adsorption capacity of Cd(II), Cu(II) and Zn(II) with other biochar.

Metal	Feedstock	Experiment Conditions				Q_{max} (*mg·g^{-1}*)	Reference
		Pyrolysis Temperature	Initial pH	C_0 (*mg*·L^{-1})	Sorbent Dosage (*g*·L^{-1})		
Cu	Spent Agaricus bisporus substrate	750	5	0–250	1	68.1	Current study
	Jarrah	700	5.5	0–193	1.25	4.39	1
	Corn straw	800				25.6	2
	Algae-dairy-manure	600	6	5–300	2	14.83	3
	Cattle manure	500	2	2–50	5	44.50	4
	Cauliflower leaves	600	-	50–200	2.5	53.96	5

	corn straw	600	5	6–318	5	12.52	6
Zn	Spent Agaricus bisporus substrate	750	5	0–250	1	55.2	Current study
	Rice straw	550	5	0–65	1.25	39.69	7
	Chicken manure	550	5	0–65	1.25	11.24	7
	Sewage sludge	550	5	0–65	1.25	4.25	7
	Jarrah	700	5	0–327	5	2.31	1
Cd	Spent Agaricus bisporus substrate	750	5	0–250	1	64.8	Current study
	Bamboo	700	5	2–300	1	76.18	8
	Pine needle	600	6	2.5–360	2	53.8	9
	Municipal sewage sludge	550	7	10–50	5	41.67	10

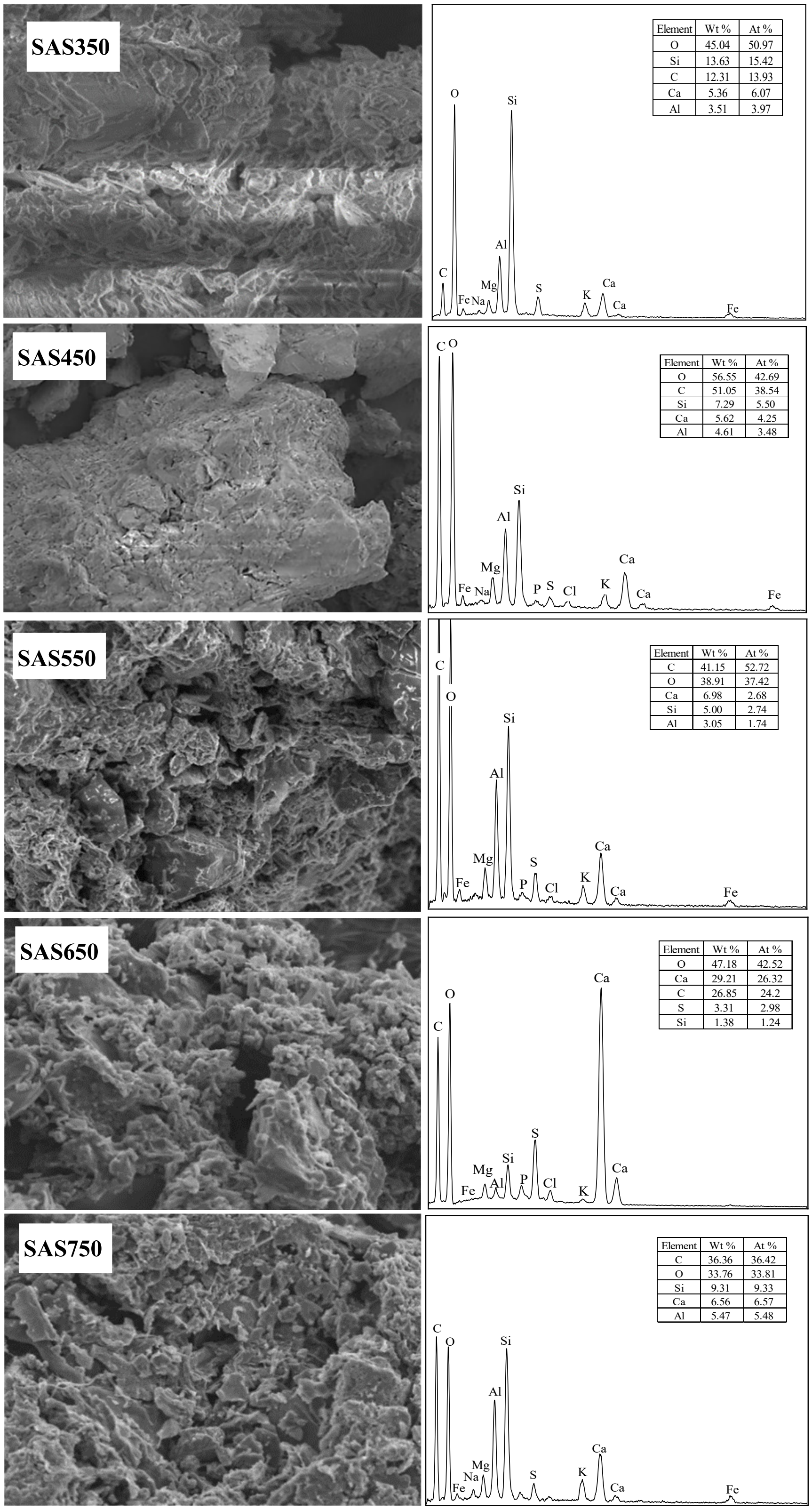

Figure S1. The SEM images (3000×) and corresponding EDS spectra of spent *Agaricus bisporus* derived biochars at 350–750 °C. SAS300–SAS700 were the biochars at 350–750 °C.

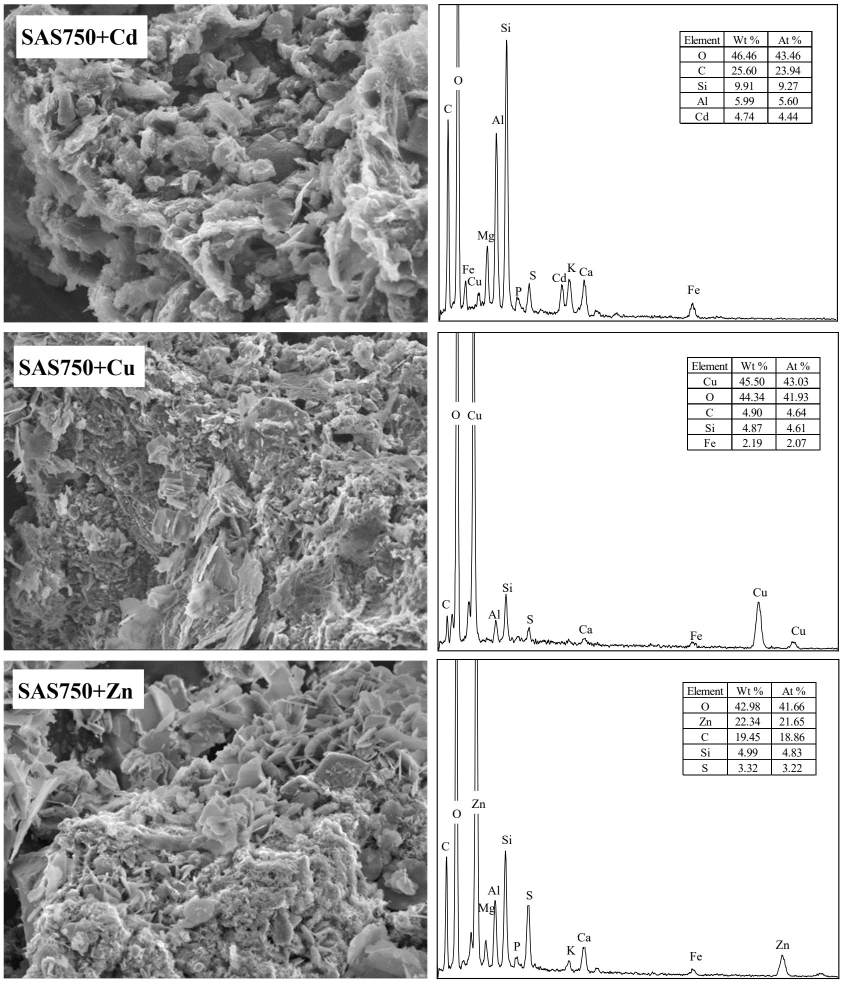

Figure S2. The SEM images (3000×) and corresponding EDS spectra of spent *Agaricus bisporus* derived biochars after adsorption of Cd(II), Cu(II) and Zn(II) at 750 °C.

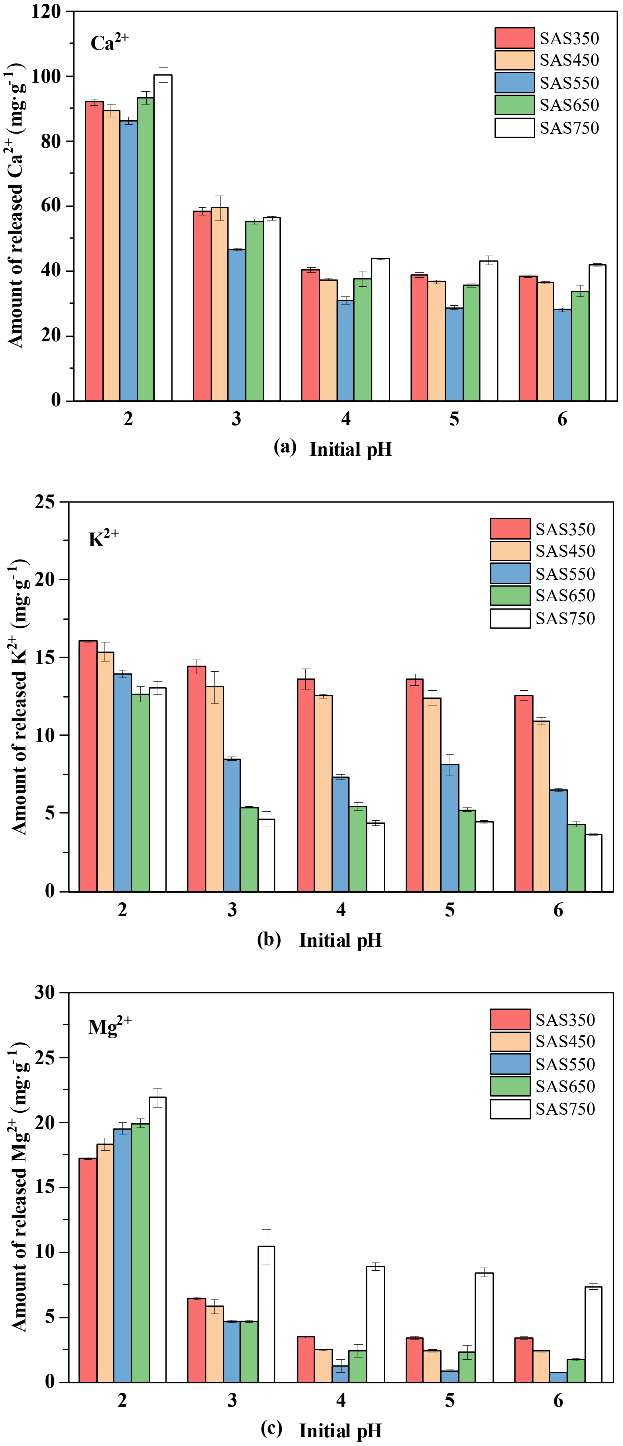

Figure S3. The amount of Ca^{2+} (**a**), K^{+} (**b**) and Mg^{2+} (**c**)released from SABCs into solution after Zn(II) adsorption at different initial pH values. (pH of 5, 24 h, initial concentration of 100 mg·L^{-1} and adsorbent dosage of 1 g·L^{-1}).

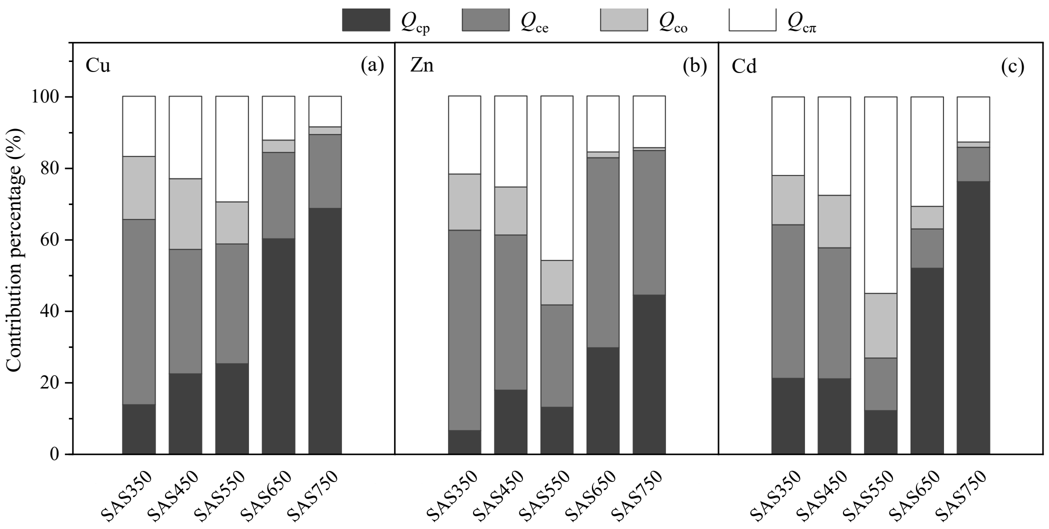

Figure 4. The contribution percentage of different mechanisms to Cu(II) (**a**), Zn(II) (**b**) and Cd(II) (**c**) sorption on SASCs. (pH of 5, 24 h, initial concentration of 100 mg·L^{-1} and adsorbent dosage of 1 g·L^{-1}).

References

1. Jiang, S.; Huang, L.; Nguyen, T. A. H.; Ok, Y. S.; Rudolph, V.; Yang, H.; Zhang, D. Copper and zinc adsorption by softwood and hardwood biochars under elevated sulphate-induced salinity and acidic pH conditions. *Chemosphere* **2016,** *142*, 64–71.
2. Yu, W.; Lian, F.; Cui, G.; Liu, Z. N-doping effectively enhances the adsorption capacity of biochar for heavy metal ions from aqueous solution. *Chemosphere* **2018,** *193*, 8–16.
3. Jin, H.; Hanif, M. U.; Capareda, S.; Chang, Z.; Huang, H.; Ai, Y. Copper(II) removal potential from aqueous solution by pyrolysis biochar derived from anaerobically digested algae-dairy-manure and effect of KOH activation. *Journal of Environmental Chemical Engineering* **2016,** *4*, 365–372.
4. Idrees, M.; Batool, S.; Kalsoom, T.; Yasmeen, S.; Kalsoom, A.; Raina, S.; Zhuang, Q.; Kong, J. Animal manure-derived biochars produced via fast pyrolysis for the removal of divalent copper from aqueous media. *Journal of Environmental Management* **2018,** *213*, 109–118.
5. Ahmad, Z.; Gao, B.; Mosa, A.; Yu, H.; Yin, X.; Bashir, A.; Ghoveisi, H.; Wang, S. Removal of Cu(II), Cd(II) and Pb(II) ions from aqueous solutions by biochars derived from potassium-rich biomass. *Journal of Cleaner Production* **2018,** *180*, 437–449.
6. Chen, X.; Chen, G.; Chen, L.; Chen, Y.; Lehmann, J.; McBride, M. B.; Hay, A. G. Adsorption of copper and zinc by biochars produced from pyrolysis of hardwood and corn straw in aqueous solution. *Bioresource Technology* **2011,** *102*, 8877–8884.
7. Zhao, M.; Dai, Y.; Zhang, M.; Feng, C.; Qin, B.; Zhang, W.; Zhao, N.; Li, Y.; Ni, Z.; Xu, Z.; Tsang, D. C. W.; Qiu, R. Mechanisms of Pb and/or Zn adsorption by different biochars: Biochar characteristics, stability, and binding energies. *Science of The Total Environment* **2020,** *717*, 136894.
8. Wang, R. Z.; Huang, D. L.; Liu, Y. G.; Zhang, C.; Lai, C.; Zeng, G. M.; Cheng, M.; Gong, X. M.; Wan, J.; Luo, H. Investigating the adsorption behavior and the relative distribution of Cd(2+) sorption mechanisms on biochars by different feedstock. *Bioresour Technol* **2018,** *261*, 265–271.
9. Park, J.-H.; Wang, J. J.; Kim, S.-H.; Kang, S.-W.; Jeong, C. Y.; Jeon, J.-R.; Park, K. H.; Cho, J.-S.; Delaune, R. D.; Seo, D.-C. Cadmium adsorption characteristics of biochars derived using various pine tree residues and pyrolysis temperatures. *Journal of Colloid and Interface Science* **2019,** *553*, 298–307.
10. Chen, T.; Zhou, Z.; Han, R.; Meng, R.; Wang, H.; Lu, W. Adsorption of cadmium by biochar derived from municipal sewage sludge: Impact factors and adsorption mechanism. *Chemosphere* **2015,** *134*, 286–293.
